# Sequential delivery of therapeutic agents using a rationally designed disulfide-linked glycolipid-like nanocarrier

**DOI:** 10.18632/oncotarget.13083

**Published:** 2016-11-04

**Authors:** Yingwen Hu, Na Liu, Bolin Cheng, Yanan Tan, Lijuan Wen, Hong Yuan, Fuqiang Hu

**Affiliations:** ^1^ Institute of Pharmaceutics, College of Pharmaceutical Sciences, Zhejiang University, Hangzhou 310058, People's Republic of China; ^2^ Department of Chemistry, Purdue University, West Lafayette, IN 47907, United States of America

**Keywords:** co-delivery, sequential, combined therapy, redox-responsive, drug resistance

## Abstract

Usage of combination therapies to deliver multiple therapeutics to increase treatment efficacy has shown promising results in the clinic. In an effort to maximize the synergistic effect of co-delivery of a drug and siRNA, we have developed a time-dependent sequential drug delivery system (DDS) based on a disulfide-linked chitosan-based nanocarrier (CS-ss-SA) for the co-delivery of paclitaxel (PTX) and Bcl-2 specific siRNA (siBcl-2). This CS-ss-SA nanocarrier is able to transport both drug and siRNA by entrapment of PTX and adsorption of siRNA on the shell by electrostatic attraction. We show that this nanocarrier transports siRNA into tumor cells via its glycolipid-like spatial structure and releases a hydrophobic model drug, Nile Red 8-11 h later. Next, when siRNA and the hydrophobic drug PTX were co-delivered to tumor cells, a synergistic effect was observed in both cell cycle arrest and cell viability. Ultimately, the co-delivery of PTX and siBcl-2 by CS-ss-SA may prove to be more efficacious and may even help overcome drug resistance.

## INTRODUCTION

In recent years, advances in nanotechnology and biotechnology have opened up unprecedented opportunities for controlled drug delivery and novel co-delivery strategies [1, 2]. With a rational design, a drug combination with different therapeutic agents in the same delivery system can allow significant benefits in cancer therapy, including reduced therapeutic doses, side effects, development of drug-resistance and, in the long run, cost [3–6]. In particular, through gene silencing, a window can open where the tumor cells are transiently sensitized to a cytotoxin. Therefore, sequential delivery of siRNA and a conventional cytotoxin in a single nanocarrier offers considerable potential for improving therapeutic outcomes [7–10].

However, it should be noted that the chemotherapeutics and RNAi combination therapy still faces significant challenges to fulfill potential clinical applications. First, the optimized timing/sequence of release of the cytotoxic and siRNA agents from the system, which is a key factor to achieve the best synergistic outcome is not well understood. The ideal co-delivery carrier with an optimized drug releasing sequence should first release the siRNA to achieve knock-down of the target gene, leading to a transient window of increased cytotoxin sensitivity. The ideal co-delivery carrier would only then release the cytotoxin, for maximum efficacy [11, 12].

According to these principles, we proposed a disulfide-linked glycolipid-like nanocarrier (chitosan-SS-stearic amine, CS-ss-SA) as the co-delivery system. Chitosan, with a positive zeta potential has been demonstrated to compact oligonucleotides readily on its shell [13]. With the particular spatial structure of the glycolipid-like nanocarrier, hydrophobic drugs can be easily encapsulated in their inner cores, which makes it possible to deliver both nucleotide and small molecule drugs in one regimen [14, 15]. With the selective redox responsive disulfide linker, the shell will be detached upon internalization, releasing the compacted siRNA. Subsequently, the cytotoxin will gradually release from the core into the cytoplasm, exerting its cytotoxic effects. Therefore, this nanocarrier would allow for the sequential delivery of therapeutic agents.

**Scheme 1:**
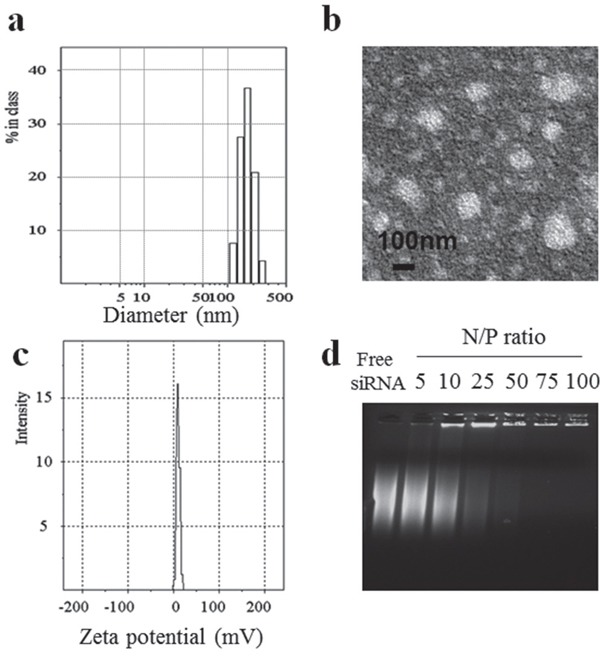
The schematic diagram of the co-delivery system Schematic structure of siRNA targeted to Bcl-2 (siBcl-2) and paclitaxel (PTX) loaded CS-ss-SA complexes, which was fast uptake by tumor cells, responded to the endogenous high GSH in tumor cells and sequentially released the siRNA and PTX to perform maximized cytotoxicity.

In this manuscript, we report the development of a glycolipid-like nanocarrier based on chitosan (CS-ss-SA) for the co-delivery of a Bcl-2 specific siRNA (siBcl-2) and the hydrophobic antitumor drug paclitaxel (PTX). We studied the cellular pathway of the co-delivery system, including internalization, endo-lysosome escape and sequential drug release against the MCF-7 breast cancer cell line. Then we investigated cell cycle arrest and loss of cell viability induced by the co-delivery of PTX and siBcl-2 to normal and multi-drug resistant MCF-7 cell lines. We demonstrate time dependent sequential drug delivery achieves synergetic effects and potentially can be used to treat drug resistant cell lines (Scheme 1).

## RESULTS AND DISCUSSION

### Preparation and characterization of co-delivery core/shell complexes

The nano-carrier CS-ss-SA was prepared using a previously reported procedure [16]. SA was crosslinked with chitosan by a two-step amide coupling to produce CS-ss-SA. The amino substitution ratio (SD%) was determined as 9.57% (molar ratio), which provided the molecular weight of the final polymer as approximately 18.9 KDa.

**Figure 1:**
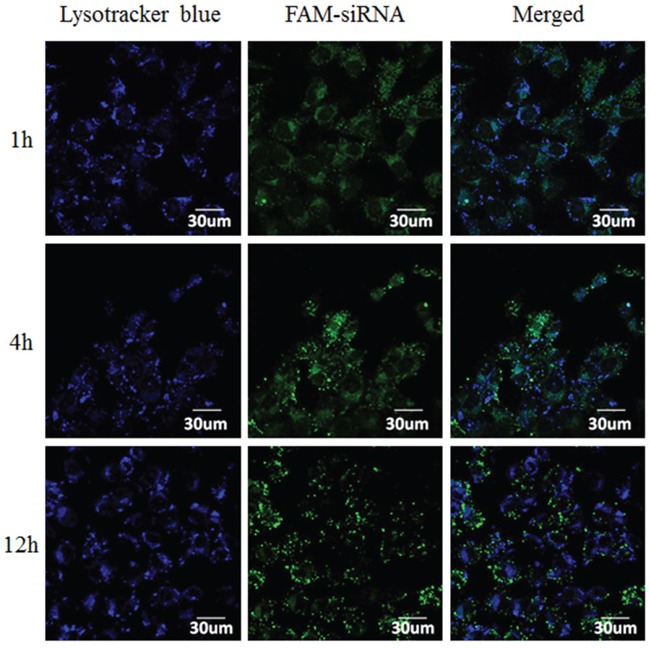
Characteristics of CS-ss-SA/PTX/siRNA complexes **a.** size distribution. **b.** TEM observation, **c.** zeta potential, **d.** gel retardation analyses of complexes with different N/P ratios.

To prepare the disulfide-linked glycolipid-like co-delivery complexes, first, paclitaxel was loaded into Chitosan-SS-steraic acid (CS-ss-SA) with approximately 80% loading efficiency. Next, siRNA was electrostatically bound to the positive amine groups on the chitosan shell of the drug-loaded nanoparticles. The hydrodynamic size of the co-delivery core/shell nanoparticles was measured to be 160.0 ±37.0 nm by DLS (Figure 1a) and the size measured by TEM was approximately 140 nm (Figure 1b), which showed increased particle size than only drug-loaded nanoparticles (Figure S1). The zeta potential of the co-delivery complexes was 15.6 ± 2.8 mV (Figure 1c), which was lower than that of only drug-loaded nanoparticles (33.9 ± 1.3 mV) because of the incorporation of negatively charged siRNA on the shell. The binding ability of the complexes was assessed using a gel retardation assay (Figure 1d). When the N/P ratio of complexes reached 75, the migration of siRNA was completely retarded, suggesting that stable complexes were formed when the N/P ratio was at or above 75. Since siRNA is readily enzymatically degraded by RNases, a critical advantage of nanocarrier-mediated delivery is the ability to prevent premature destruction of siRNA [14]. In order to assess the capacity of CS-ss-SA to protect siRNA from degradation by RNases, CS-ss-SA/siRNA complexes were prepared with N/P ratios = 75 and incubated with a solution of RNase A. Degradation of siRNA was visualized via gel electrophoresis and the results are presented in Figure S2. Free siRNA was completely digested with no full-length band observed, while clear migration of the full-length siRNA band was observed even after RNase treatment. These results indicate that CS-ss-SA is able to effectively protect siRNA from RNase degradation.

### *In vitro* siRNA release

In order to release the siRNA from the chitosan shell, the disulfide bonds must be reduced to free thiols, which disrupt the overall structure of the DDS. In order to tune the sensitivity of the disulfide reduction such that the siRNA is not prematurely released before the DDS reaches its target, the structural characteristics of the DDS were modified. In previous studies, we have prepared CS-ss-SA with a range of graft ratios [16]. We found that the introduction of additional disulfide linkers decreased the sensitivity to cleavage in higher reducing environments. Using this rational design strategy, CS-ss-SA could degrade correspondingly to different levels of reducing environment, release the payloads, and be used for triggered-release in different tumor types. In this study, we used the optimal ratio of disulfide linkers to construct the CS-ssSA_20%_ which should only release their siRNA cargo in highly reducing environments. To determine the rate of release, fluorescently labeled siRNA was adsorbed onto CS-ss-SA_20%_ nanoparticles. These nanoparticles were incubated in non-reducing and reducing (10 mM GSH) environments. As shown in Figure S3, fluorescence intensity (I) and FAM-siRNA concentrations (C) showed a linear regression: I = 37.08 × C – 0.62, r^2^ = 0.9995, which confirmed to be 0.165–13.2 μg/mL. The disassociation of the siRNA from the CS-ss-SA/FAM-siRNA complex was fairly rapid with a cumulative release of 78.2% in 8 h, when in the presence of 10 mM GSH. While in the GSH-free release medium, siRNA release rate was relatively slow in which the cumulative release was less than 50% at 12h. The difference in release rates is most likely due to the weakening of the electrostatic interactions between chitosan and siRNA upon reduction of the nanoparticle chemical backbone.

### Cellular uptake and endo-lysosome escape

**Figure 2:**
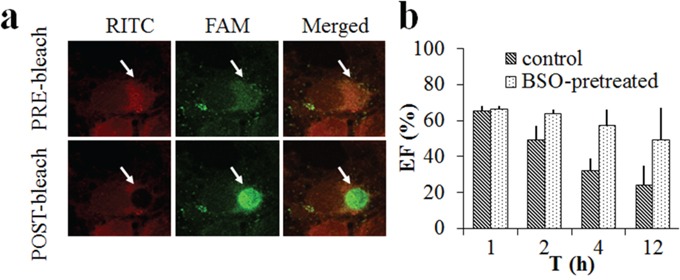
Confocal images of intracellular trafficking of the complexes and lysosome on MCF-7 cells Lysotracker-labelled lysosomes (blue), FAM-labelled siRNA (green), and the merged images.

To track complexes after cellular uptake and to evaluate their endo-lysosome escape capacity, the intracellular distribution of CS-ss-SA/siRNA complexes in MCF-7 cells was investigated by confocal laser scanning microscopy (CLSM) (Figure 2). Co-localization of the fluorescent FAM-siRNA (green) with Lysotracker-stained lysosomes (blue) produced cyan fluorescence in the merged images. At 1h, large numbers of cyan pixels were observed in treated cells with 82.4% of siRNA co-localized with lysosomes. This result indicates the CS-ss-SA/siRNA complexes were trapped in the lysosomes. After 4 h, fewer cyan pixels were observed. After 12 h, only 8.03% of siRNA was co-localized with lysosomes, suggesting that most of the complexes have escaped. These observations may be due to the ionization state of chitosan at tumor acidic endo-lysosome environment (pH 4.5 – 6.4), which is near its pKa (5–7 for chitosan and independent on the degree of ionization), which may increase endosomal pH leading to endosomal release [13, 21].

### Intracellular triggered siRNA release

To further characterize the intracellular siRNA release, a FRET assay and molecular beacons (MB) were applied. Real-time monitoring of redox-responsive complexes in the presence of high/low levels of GSH in MCF-7 cells was enabled.

In this study, non-coding siRNA labeled with FAM (FAM-siNC, E_x_=487 nm) and the cationic vector CS-ss-SA labelled with RITC (RITC-CS-ss-SA, E_x_=546 nm) were used as the FRET donor and receptor, respectively. These two dyes were chosen because of their superior photostability, large spectrum overlaps and high FRET efficiency.

**Figure 3:**
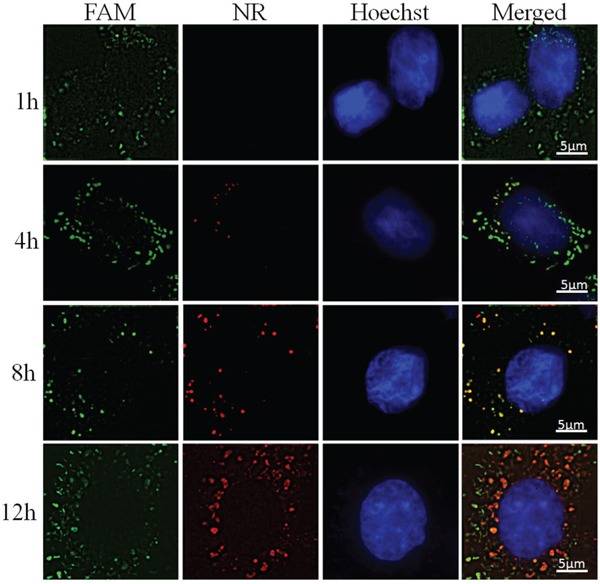
FRET images on the MCF-7 cells incubated with RITC-labelled CS-ss-SA/FAM-siRNA complexes **a.** FRET images before and after acceptor bleaching in fixed cells, from left to right: RITC-labeled CS-ss-SA, FAM-labeled siRNA, and merged channel. **b.** Quantitative determination of FRET_eff_ on the BSO-pretreated or untreated MCF-7 cells in 12 h.

Buthionine sulfoximine (BSO) was added to lower the intracellular GSH concentration according to literature and untreated MCF-7 cells were used as a control [22]. RITC-CS-ss-SA/FAM-siRNA complexes were incubated with BSO pre-treated or untreated cells. FRET_eff_ (%) between the donor and acceptor as a function of time is shown in Figure 3. When the receptor (RITC) was bleached in the region, fluorescence intensity of the donor (FAM, green) rapidly increased, indicating significant FRET between the two fluorophores (Figure 3a). As seen in Figure 3b, after 1 h of incubation, the different GSH concentrations made no significant difference in the FRET_eff_ of the MCF-7 cells. In 2 h, the FRET_eff_ of untreated MCF-7 cells decreased significantly (p = 0.032). After 12 h of incubation, the FRET_eff_ of untreated MCF-7 cells decreased even further to below 30%, indicating siRNA release from the complexes during the 12 h. Whereas the FRET_eff_ of BSO pre-treated cells maintained a high percentage (~50%), which illustrated that the release of siRNA from the complexes was significantly slower with a lower GSH concentration in the BSO pre-treated cells.

Molecular beacon (MB) is a dual-labeled hairpin oligonucleotide probe comprising a fluorophore and a quencher at opposite ends. With the stem-loop structure, the fluorescence of the fluorophore remains quenched in the absence of a complementary target. When the molecular beacon hybridizes with its target, the hairpin structure will open, allowing the fluorescence signal to be emitted [23, 24]. Based on these principles, a Cy5-labeled molecular beacon targeting the human GAPDH (glyceraldehyde 3-phosphate dehydrogenase) GAPDH-MB (Cy5-MB) was utilized in this study to transduce the oligonucleotide release from the CS-ss-SA/Cy5-MB complexes directly into a fluorescence signal by flow cytometry.

Fluorescence intensity of untreated and BSO-pretreated MCF-7 cells as a function of time was shown in Figure S4. The fluorescence intensity of untreated group increased gradually over time, which indicated that with the high GSH concentration in the tumor cells, Cy5-MB fast released from the CS-ss-SA/Cy5-MB complex, bind to the target GAPDH sequence, and emitted fluorescence. Whereas in the BSO-pretreated group, intracellular fluorescence intensity showed no significant change over time. The fluorescence intensity of the untreated group was 39 times higher than that of the BSO-pretreated group, indicating that the amount of free MB in the BSO-pretreated cells dissociated from the complex was only 2.6% of the untreated group.

These results demonstrated that in response to the high concentration of reducing agents, CS-ss-SA complexes can readily release the siRNA/oligonucleotides into tumor cells. When the GSH concentration was decreased by BSO in the tumor cells, the release rate significantly decelerates.

### Intracellular sequential drug delivery

The biggest challenge in co-delivery is to find applicable carriers to optimize the timing/location of release of chemotherapy and siRNA agents in tumor cells. The incorporation of additional drug payloads affects the pharmacokinetics of the nanocarrier and requires significant modifications of the carrier design. We further studied the intracellular sequential drug delivery of complexes in MCF-7 cells. Nile red (NR) and FAM-labeled non-coding siRNA (siNC) were used as the model drugs and fluorescent probes.

NR was chosen for its unique spectral signature, where the emitted signal is dependent upon the hydrophobicity of its environment. When NR became trapped inside the nanoparticle, its fluorescence intensity at this wavelength essentially disappeared (Figure S5).

**Figure 4:**
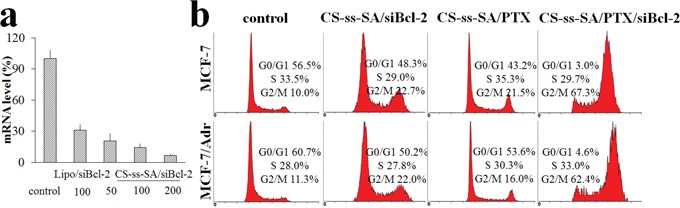
Sequential delivery FAM-siNC and NR in MCF-7 cells after incubated with CS-ss-SA/NR/FAM-siNC complexes for 1, 4, 8, and 12 h From left to right, FAM-labeled non-coding siRNA, Nile Red, Hoechst labeled nuclei and merged channel.

The cells underwent incubation with CS-ss-SA/NR/FAM-siNC complexes for periods of 1, 4, 8, and 12 h respectively and intracellular fluorescence images were observed to monitor the drug delivery behavior. As shown in Figure 4, large numbers of green fluorescent bodies (FAM-siNC) were observed in the first hour, indicating the complexes first released the siRNA cargo nearly immediately after cellular internalization. The majority of visible red fluorescent bodies (NR) started to appear after the 8 h timepoint, which shows the distinctly different release kinetics of the two cargoes with the NR clearly being released 7 to 11 hours later than the siRNA.

### *In vitro* siRNA transfection

**Figure 5:**
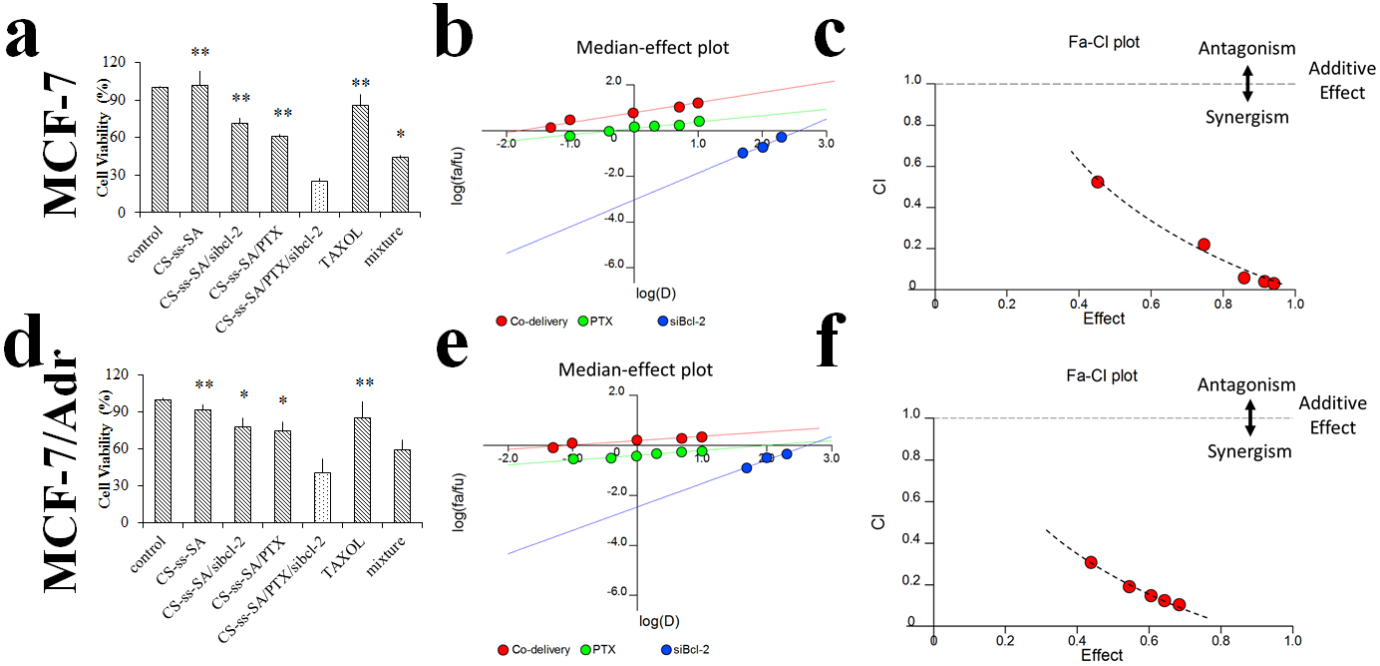
**a.** Bcl-2 mRNA levels regulation in MCF-7 cells by the transfection of CS-ss-SA/siBcl-2. **b.** Cell cycle distributions of MCF-7 and MCF-7/Adr cells incubated with CS-ss-SA/siBcl-2, CS-ss-SA/PTX and CS-ss-SA/PTX/siBcl-2 complexes (siBcl-2 100 nM, PTX 0.1 μg/mL) for 48 h.

MCF-7 cells were incubated with CS-ss-SA/siBcl-2 complexes for 24 h and then intracellular Bcl-2 mRNA levels were determined by RT-PCR. After sequence-specific Bcl-2 silencing by CS-ss-SA/siBcl-2 complexes, reduced Bcl-2 mRNA levels were observed (Figure 5a). Elevated siBcl-2 concentrations produced increased gene knockdown, with 50, 100 and 200 nM of siBcl-2 by CS-ss-SA/siBcl-2 complexes inducing 79.4%, 85.8% and 93.1% knockdown of Bcl-2 mRNA. In comparison, the negative control showed neglible knockdown efficiency. Transfection using the CS-ss-SA/siBcl-2 complexes is similar to that of the Lipofectamine^2000^ transfection reagent carrying the same dose, indicating that the gene vector is highly effective.

### Cell cycle analysis

Overexpression of Bcl-2 protein is related to drug resistance and poor prognoses in cancer patients [25]. Simply knocking down Bcl-2 has not been found to effectively suppress cancer activity, most likely due to the complexity of the apoptotic signaling pathway [26–29]. According to the mechanism of drug action, the silencing of the Bcl-2 gene will open a window of time in which the cell population's cell cycle is transiently synchronized, becoming more sensitive to chemotherapy [11, 30]. As one of the most frequently used drugs in the treatment of breast cancer, dose-dependent side effect of PTX greatly limits its clinical application [31]. Thus, reducing the PTX dose or increasing the sensitivity of tumor cells is of particular importance. PTX interferes with mitotic spindle function through tubulin polymerization and arrests the G2/M phase of cell cycle.

In this study, CS-ss-SA/siBcl-2/PTX complexes were incubated with MCF-7 cells and multi-drug resistant MCF-7/Adr cells, which introduced G2/M cell-cycle arrest after 48 h (Figure 5b). In MCF-7 cells, G2/M population gradually increased from 10.0% to 21.5%, indicating that PTX-loaded CS-ss-SA nanoparticles release PTX. Whereas after incubation with CS-ss-SA/siBcl-2/PTX complexes, the G2/M population increased significantly to 67.3%, indicating that co-delivery of siBcl-2 and PTX was more efficacious. A similar tendency was also seen in MCF-7/Adr cells, indicating the possibility of the synergetic effect induced by the co-delivery system against both drug-sensitive and resistant cells.

### Synergy quantification of the co-delivery complexes

**Figure 6:**
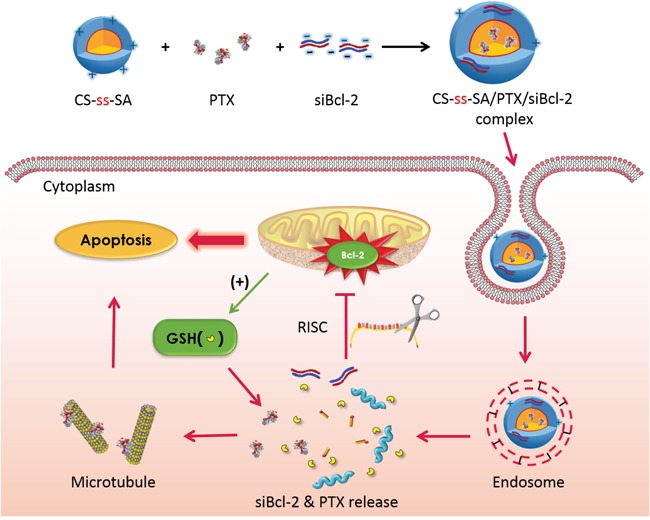
Cytotoxicity of the co-delivery complexes **a, d.** Cell viability of the co-delivery of siBcl-2 and PTX by CS-ss-SA against MCF-7 and MCF-7/Adr cells. The concentration of PTX was 0.1 μg/mL, while the concentration of siBcl-2 was 100 nM. **b, e.** Median-effect plot. “D” represents the dose (or concentration) of a drug, fa is the fraction affected by D, and fu is the fraction unaffected (i.e., fu = 1 - fa). Dm is the median-effect dose (IC_50_ in this study) that inhibits the system under study by 50%, and m is the coefficient signifying the shape of the dose-effect relationship, where m = 1, > 1, and < 1 indicate hyperbolic, sigmoidal, and flat sigmoidal dose-effect curves, respectively. **c, f.** Plot of the combination index (CI) as the function of cell viability. Untreated cells were used as control. The y-axis (CI) was a function of effect levels (fa) on the x-axis. CI was introduced for quantification of synergism or antagonism for two drugs, where CI < 1, = 1, and > 1 indicate synergism, additive effect, and antagonism, respectively.

Cytotoxicitystudies were performed to probe the potential synergistic effect of the co-delivery system to downregulate the apoptosis threshold and overcome the drug resistance of MCF-7 and MCF-7/Adr cells. The experiments were designed to gain insight into the quantitative measurement of Bcl-2 silencing and PTX to exert their maximum effect. As shown in the Figure 6a-6c, CS-ss-SA micelles exhibited negligible toxicity in MCF-7 cells, while CS-ss-SA/siBcl-2/PTX complexes showed a high cytotoxicity (74.9%). The co-delivery CS-ss-SA/siBcl-2/PTX complexes presented significantly higher cytotoxicity than that of CS-ss-SA/PTX nanoparticle (39.1%, p = 0.0023) or CS-ss-SA/siBcl-2 complexes (28.6%, p = 0.0082) alone. Since the concentration of PTX in the experiment was 0.1 μg/mL, the greatly increased cytotoxicity was possibly attributed to the synergistic effect of PTX and silencing of Bcl-2 gene.

To further confirm the synergistic assumption of the co-delivery system, we used the the Chou-Talalay combination index (CI) method to quantitate the synergistic effect [32]. A free computer software “Calcusyn” provided by Prof. Chou was used for data analysis. The software generated the simulation and provided PD parameters (Dm and m), curves for dose-effect, the Fa-CI and median effect plot, and the Summary Table. The CI values of the co-delivery system as a function of the cell viability was simulated (Figure 6). In Figure 6b and 6e, 6D represents the drug dose, fa is affected fraction, while fu is the unaffected fraction. Dm represents the half maximal inhibitory concentration (IC_50_), and m is the coefficient. CI was introduced for quantification of synergistic or antagonistic effects for the two drugs:

$$\begin{array}{lll} \text{CI} & = & \frac{{\left(D\right)}_{1}}{{{(D}_{x})}_{1}}+\frac{{\left(D\right)}_{2}}{{\left(D_{x}\right)}_{2}}=\frac{{\left(D\right)}_{1}}{{\left(D_{m}\right)}_{1}\;f_{a}/{\left(1-f_{a}\right)}^{1/m_{1}}}+\\ & & \frac{{\left(D\right)}_{2}}{{\left(D_{m}\right)}_{2}\;f_{a}/{\left(1-f_{a}\right)}^{1/m_{2}}} \end{array}$$

where CI < 1 or > 1 indicate a synergistic or antagonistic effect, respectively.

In this study, calculated CI values were established for doses providing a 50, 75, and 90% cancer cell-killing effect (ED_50_, ED_75_ and ED_90_). The results for ED_90_ revealed that co-delivery of PTX and siBcl-2 with all doses had CI values between 0.29 to 0.03, indicating a strong synergistic effect (Figure 6c). For the drug resistant MCF-7/Adr cells, the CI values ranged from 0.31 to 0.10, also indicating a significant synergism (Figure 6f). Based on these results, we conclude that the co-delivery complexes had synergistic effects, and showed the capacity of overcoming multi-drug resistance.

Interestingly, the co-delivery system showed higher cell growth inhibition than that of the mixture of PTX-loaded nanoparticles and siRNA-bound complexes administered at the same dose. Reports have shown that siRNA and chemotherapeutic agents co-delivered in the same carrier system had a greater inhibitory effect on cell proliferation than delivered separately in different carriers [33–35]. This is mainly because the same drug delivery system with chemotherapeutics and siRNA guaranteed similar cell internalization and intracellular sequential drug release for both treatments in the tumor cells. For long-term consideration, a co-delivery system with the same carrier could guarantee similar pharmacokinetics and concomitant passive accumulation of both treatments in the same tissue and cells [1, 36].

## CONCLUSION

In conclusion, we have designed and applied an effective co-delivery nanocarrier for an optimized synergetic effect. Significantly, the release of siRNA was correlated positively with the intracellular GSH level. SiRNA and a hydrophobic drug were sequentially delivered into cells by a nanocarrier and generated cell cycle arrest and cytotoxicity in breast cancer cells and drug-resistant cells. This co-delivery system will facilitate the rational design of a sequential combined therapy.

## MATERIALS AND METHODS

### Materials

Chitosan with an approximate 15.0 kDa average molecular weight was obtained by enzymatic degradation of 95% deacetylate chitosan (Mw = 450 kDa) and was supplied by Yuhuan Marine (Yuhuan, China). Stearic acid (SA) was purchased from Fluka (Milwaukee, WI, USA). 3, 3′-dithiodipropionic acid was purchased from Tokyo Chemical Industry (Tokyo, Japan). FAM-siRNA (FAM-5'-UUCUCCGAACGUGUCACGUTT-3), Non-coding siRNA (siNC, 5′-UUCUUCGAACGUGUCACGUTT-3), and Bcl-2 specific siRNA (siBcl-2, 5′-CCCUGUGGAUGACUGAGUATT-3) was purchased from Shanghai GenePharma (Shanghai, China). Paclitaxel (PTX) was purchased from Shanghai Zhongxi Sunve (Shanghai, China). Cell Cycle Detection Kit was obtained from Keygene Biotech (Nanjing, China). L-Glutathione (GSH) and Nile red (NR) were purchased from Sigma-Aldrich (Diegem, Belgium). Fluorescein isothiocyanate (FITC), Rhodamine B Isothiocyanate (RITC), and buthionine sulfoximine (BSO) were purchased from Sigma (St. Louis, MO, USA). Cy5 labeled GAPDH molecular beacon (Cy5-GAPDH-MB, 5′-Cy5-CGACGGAGTCCTTCCACGATACCACGTCG-Dabcyl-3′) was purchased from Sangon Biotech (Shanghai, China). Lipofectamine™ 2000 and LysoTracker®Blue DND.22 was purchased from Invitrogen (Carlsbad, US). All of the other chemicals were of analytical or chromatographic grade.

### Cell culture

MCF-7 (human breast carcinoma cell line) and MCF-7/Adr (multi-drug resistant variant) were provided by the Second Affiliated Hospital of Zhejiang University School of Medicine (Hangzhou, China). Cells were cultured in RMPI 1640 supplemented with 10% FBS and 100 U/ml streptomycin-penicillin. For culture of MCF-7/ADR cells, the medium was supplemented with 1.0 μg/ml ADR.

### Synthesis of CS-ss-SA

The CS-ss-SA was synthesized according to a literature method [16]. SA was conjugated with 3, 3′ -dithiodipropionic acid mediated by dicyclohexylcarbodiimide (DCC) / dimethylaminopyridine (DMAP) in a molar ratio of 1:1. The reaction was performed at 60°C for 24 h under an atmosphere of N_2_ and filtered to remove the byproducts. The intermediate product was activated by 1-ethyl-3-(3-dimethylaminopropyl) carbodiimide (EDC) and added to the chitosan aqueous solution. Anhydrous DMSO was added (DMSO: H_2_O=8:5, molar ratio) to avoid precipitation. The mixture was stirred at 60°C for the next 8 h and dialyzed against DI water for 48 h and centrifuged. The supernatant was lyophilized and rinsed using hot ethanol to remove the unreacted reagent. The product (CS-ss-SA) was re-dispersed in DI water and lyophilized.

### Loading of PTX inside the cores of CS-ss-SA

CS-ss-SA was dissolved in DI water and PTX/ethanol solution was added (20%, w/w) dropwise with constant stirring for 20 min. The mixed solution was dialyzed against DI water overnight (MWCO = 7000 Da) and the suspension was centrifuged at 4000 rpm for 10 min to remove the unloaded PTX. The resulting supernatant was lyophilized and PTX loaded CS-ss-SA was obtained. The amount of PTX inside the cores was determined by a HPLC assay [17].

### Preparation of co-delivery complexes

The CS-ss-SA/PTX/siBcl-2 co-delivery complexes were prepared by mixing the components at certain N/P ratios in DEPC-treated water. The particle size and zeta potential of the complexes were determined by DLS Spectrometer (Malvern Zetasizer).

### Gel retardation studies

For gel retardation studies, CS-ss-SA/PTX/siRNA co-delivery complexes with various N/P ratios were subjected to electrophoresis on a 1.0% agarose gel containing ethidium bromide, using E-Gel electrophoresis system (Invitrogen) and visualized under UV light.

### RNase protection assay

CS-ss-SA/siRNA complexes (containing 1 μg siRNA) were incubated for 30 min at room temperature, followed by the addition of RNase A (10 U). As a control, free siRNA (1 μg) was also treated with RNase I under the same condition. After 30 min incubation, the samples were further treated with RNase inhibitor (RI, 1 μL= 40 U) and incubation for 30 min at 37°C to terminate the activation of RNase A, followed the addition of heparin (0.05%) incubated for 30 min. The integrity of siRNA was determined by gel electrophoresis at the same conditions as described before.

### Cell uptake and endo-lysosome escape studies

The *in vitro* cell uptake and endo-lysosome escape of CS-ss-SA/siRNA complexes was determined by confocal studies. Cells were seeded 24 h prior to uptake in 12-well plates containing cover slips at a density of 5×10^4^ cells per well. CS-ss-SA/siRNA complexes were added to yield a final siRNA concentration of 100 nM. FAM-labeled siRNA (FAM-siRNA) was used as the model drug. After 1, 4, and 12 h of incubation, the media were replaced and stained with Lyso-tracker. Cells were subsequently rinsed with fresh PBS and fixed with 4% paraformaldehyde (PFA). The green fluorescence of complexes and blue fluorescence of endo-lysosome were visualized with the confocal microscope (Olympus IX81-FV1000).

### Intracellular triggered siRNA release

A förster resonance energy transfer (FRET) assay was used to monitor the assembled structure and release dynamics of the complexes [18]. RITC-labeled CS-ss-SA solution was typically added to the FAM-siRNA solution at the desired N/P ratios to form FRET complexes. MCF-7 cells were seeded into 12-well plates containing cover slips at a density of 5×10^4^ cells per well. After 24 h of plating, FRET complexes (100 μL) were added. After incubation for 1, 2, 4, and 12 h, cells were rinsed with fresh PBS and fixed with 4% PFA. FRET images of complexes were visualized by confocal microscopy (Nikon A1). An acceptor bleaching (AB) method was used to measure FRET efficiency by bleaching the acceptor and calculate the enhancement of the donor fluorescence [19]. The energy transfer efficiency (FRET_eff_) was quantified as:

FRETeff=(Dpost−Dpre)Dpost×100%

Consider that molecular beacon (MB) has a unique stem–loop configuration combined with fluorophore and quencher [20], we applied a Cy5-labeled GAPDH-MB (Cy5-GAPDH-MB) as the oligonucleotide release indicator. CS-ss-SA/Cy5-GAPDH-MB complex was prepared by a similar method to the CS-ss-SA/siRNA complex. MCF-7 cells were seeded into 6-well plates at a density of 2×10^5^ cells per well. After 24 h of plating, CS-ss-SA/Cy5-GAPDH-MB complex were added. After incubation for 1, 2, 4, and 12 h, cells were rinsed with fresh PBS, collected and fixed with 4% PFA. After centrifugation at 1200 rpm for 5 min at 4°C and wash with PBS twice, the cells were re-suspended in 0.5 mL of buffer. Flow cytometry (FC500MCL, Beckman Coulter) was used to measure the fluorescence intensity.

### Intracellular sequential drug delivery trafficking

The fluorescence probe Nile red (NR) was utilized as the hydrophobic model drug and encapsulated in the CS-ss-SA/FAM-siNC complexes in accordance with the protocol. MCF-7 cells were seeded into 6-well plates containing cover slips at a density of 3×10^4^ cells per well. After 24 h of plating, sequence-release complexes (CS-ss-SA/FAM-siRNA/NR) were added. After incubation for 4h, the medium was withdrawn and replaced with fresh medium. After 1, 4, 8, and 12 h, cells were rinsed with fresh PBS and fixed with 4% PFA. Fluorescence images of complexes were visualized by structured illumination microscopy (SIM).

### Bcl-2 silencing

For gene silencing, MCF-7 cells were seeded into 6-well plates at a density of 3×10^5^ cells per well. After 24 h of plating, cells were incubated with CS-ss-SA/siBcl-2 complexes with different siBcl-2 concentrations (50, 100, 200 nM). Lipofectamine^2000^/siBcl-2 (100nM) was used for control. RNA was extracted with TRIzol according to the standard protocol and total mRNA concentrations were detected by the nanodrop spectrophotometer. Reverse transcription was conducted with PrimeScript™ RT Reagent Kit (TaKaRa) in a 20 μL SYBR® Green assay. Real-time PCR (StepOne, Applied Biosystems) was used to perform the amplification reaction. Bcl-2 primer sequence was 5′-GGATTGTGGCCTTCTTTGAG-3′ and the reverse was 5′-TACCCAGCCTCCGTTATCCT-3′. The protocol was carried out for 40 cycles, comprising 95°C for 5 s and 60°C for 34 s. GAPDH was also amplified as an internal control. A ΔΔCT method was used for relative quantification of the expression levels. Each sample was performed in triplicate.

### Cell cycle analysis

Co-delivery complexes (containing 100 nM of siBcl-2 and 100 ng of PTX) treated MCF-7 and MCF-7/ADR cells (2×10^6^ cells/well for 6-well plate) in 10% serum-containing culture medium were collected 48 h post-incubation and washed with ice cold PBS. The cells were fixed by ice cold 70% ethanol over night. After centrifugation at 1200 rpm for 5 min at 4°C and wash with PBS twice, the cells were re-suspended in 0.5 mL of buffer containing RNase A and 50 mg/mL PI. All samples were measured by Beckman Coulter Cytomics FC-500 and cell cycle distributions were analyzed.

### Cytotoxicity assay

*In vitro* cytotoxicity of the co-delivery complexes was evaluated by a cell viability assay. The MCF-7 and MCF-7/Adr cells were seeded into 96-well plates at a density of 5×10^4^ cells per well. After 24 h of plating, cells were incubated with CS-ss-SA/siBcl-2/PTX complexes. After 48h of incubation, MTT solution (5.0 mg/mL) was added and incubated for 4 h. The culture medium was changed to DMSO to dissolve the purple formazan crystals. The absorbance at 570 nm was measured using a micro plate reader. The results indicated the cytotoxicity of co-delivery complexes was calculated.

### Statistical analysis

All of the data represent the mean values ± standard deviation of the independent measurements. Statistically significant differences between pairs of mean values were determined with ANOVA followed by Tukey-Kramer tests. Average deviation with p-values < 0.05 were considered statistically significant. Differences between groups were analyzed by Student's t-test, and mean differences with p-values < 0.05 were considered statistically significant.

## SUPPLEMENTARY FIGURES


