# Supplementary material for: Sequential delivery of therapeutic agents using a rationally designed disulfide-linked glycolipid-like nanocarrier
Source: Oncotarget. 2016 Nov 4;7(50):83258–69. doi: 10.18632/oncotarget.13083 (PMC5347767; doi:10.18632/oncotarget.13083)
Supplement: Supplementary file 1 [file oncotarget-07-83258-s001.pdf]

Sequential delivery of therapeutic agents using a rationally designed disulfide-linked glycolipid-like nanocarrier

SUPPLEMENTARY FIGURES

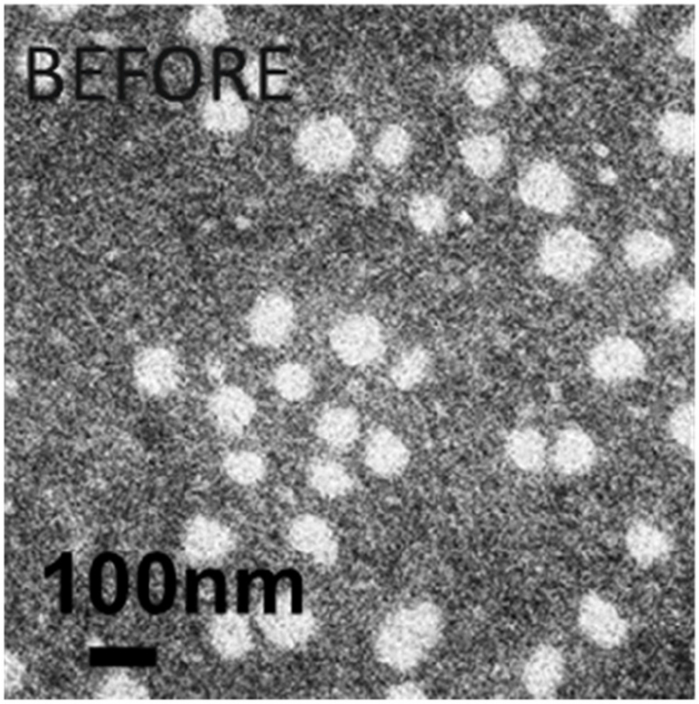

Supplementary Figure S1: TEM images before siRNA incorporation of CS-ss-SA/PTX nanoparticles.

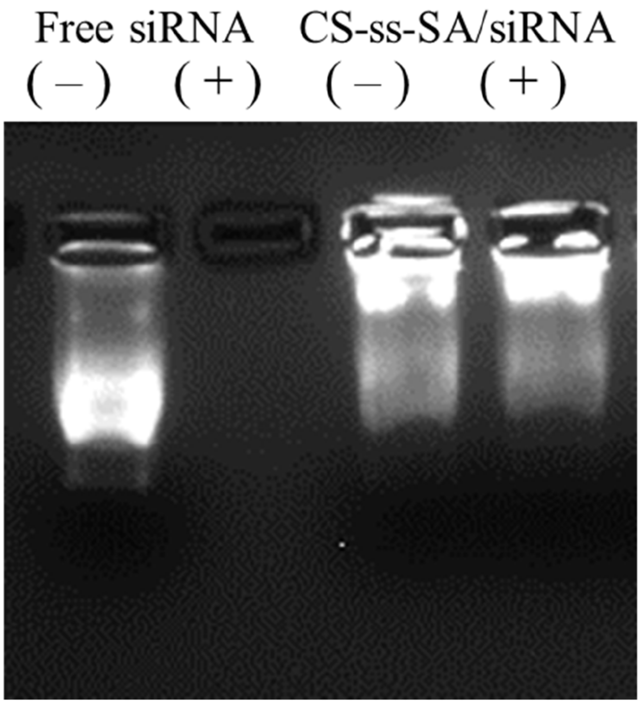

Supplementary Figure S2: RNase protection assay of the CS-ss-SA/siRNA complexes. Complexes incubated in the absence (−) or presence (+) of the RNase A treatments. Untreated siRNA control (1 µg) before (lane 1) and after (lane 2) incubation with RNase A. (Image was cropped and edited by Microsoft PowerPoint 2016 software).

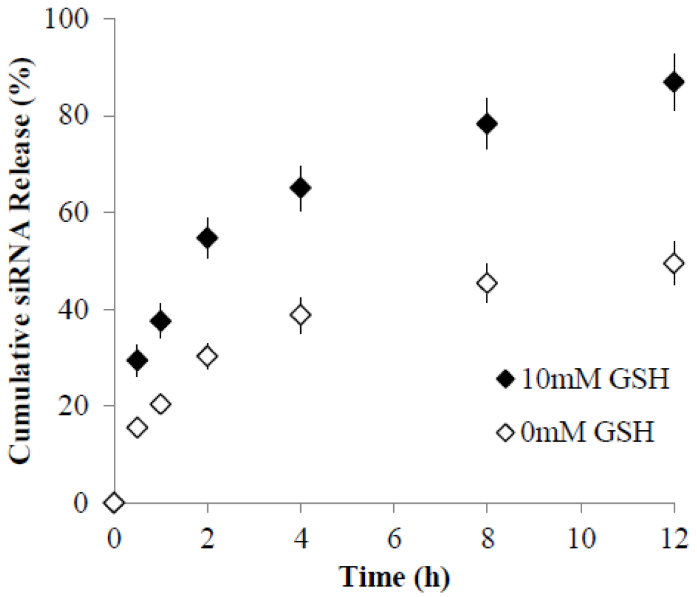

Supplementary Figure S3: Release profiles of FAM-siRNA from CS-ss-SA/siRNA complexes in Tris-HCl (0.2 mol/L, pH 7.2) supplemented with 0 and 10mM GSH. (n=3).

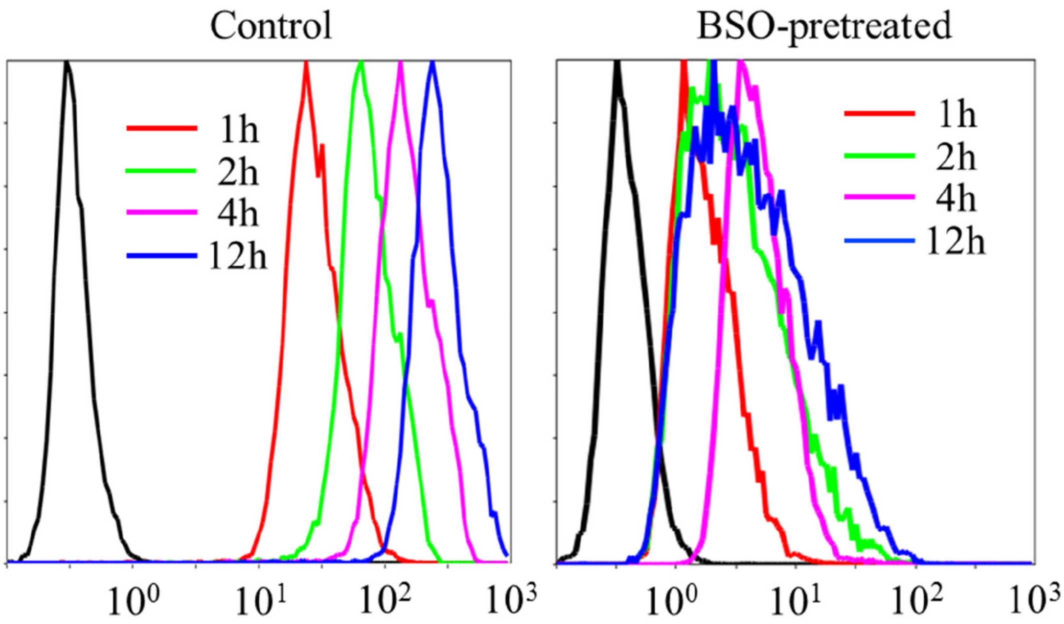

Supplementary Figure S4: Intracellular Cy5 fluorescence intensity measured by flow cytometry after the cells was incubated with the CS-ss-SA/Cy5-MB for 1, 2, 4, and 12 h. The cells were pre-treated with BSO and the untreated cells served as control.

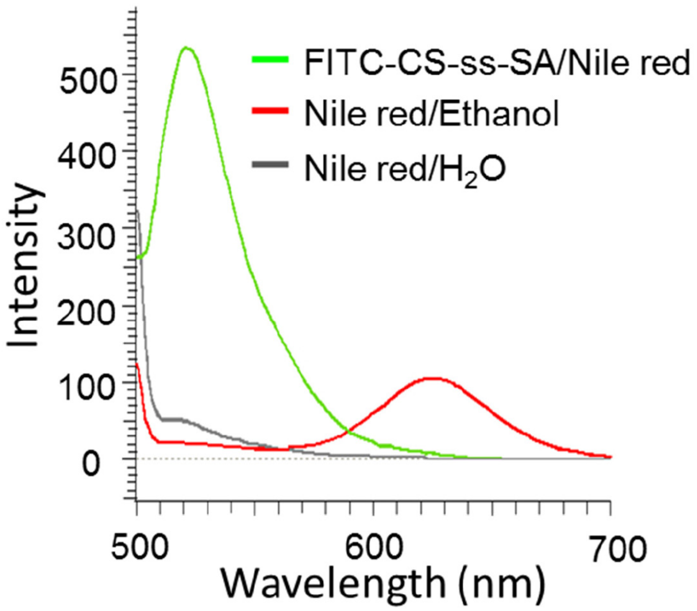

Supplementary Figure S5: Fluorescence wavelength scanning spectra of Nile red. The different colors show the Nile red entrapped in FITC-CS-ss-SA nanoparticles, dissolved in ethanol or dispersed in deionized water, respectively.
